# Primary melanoma of the oral cavity: A multi-institutional retrospective analysis in Brazil

**DOI:** 10.4317/medoral.24240

**Published:** 2020-12-19

**Authors:** Bruno Teixeira Gonçalves Rodrigues, John Lennon Silva Cunha, Danielle Mendes da Silva Albuquerque, Wagner Pinto das Chagas, Nathália de Almeida Freire, Michelle Agostini, Nathalie Henriques Silva Canedo, Ricardo Luiz Cavalcanti de Albuquerque Júnior, Silvia Ferreira de Sousa, Aline Corrêa Abrahão, Mário José Romañach, Oslei Paes de Almeida, Mônica Simões Israel, Bruno Augusto Benevenuto de Andrade

**Affiliations:** 1 DDS. School of Dentistry, Rio de Janeiro State University (UERJ), RJ, Brazil; 2 DDS, MSc student. Oral Pathology Section, Department of Oral Diagnosis, Piracicaba Dental School, University of Campinas (UNICAMP), SP, Brazil; 3 DDS, MSc. Department of Oral Diagnosis and Pathology, School of Dentistry, Federal University of Rio de Janeiro (UFRJ), RJ, Brazil; 4 DDS, MSc, Professor. São Leopoldo Mandic Dental School, Rio de Janeiro, RJ, Brazil; 5 PhD student. Department of Pathology, Fluminense Federal University (UFF), Niterói, RJ, Brazil; 6 DDS, PhD, Professor. Department of Pathology, Clementino Fraga Filho University Hospital, School of Medicine, Federal University of Rio de Janeiro (UFRJ), RJ, Brazil; 7 DDS, PhD, Professor. Oral Pathology Section, Department of Oral Diagnosis, Piracicaba Dental School, University of Campinas (UNICAMP), SP, Brazil; 8 DDS, PhD, Professor. Department of Dentistry, Tiradentes University (UNIT), Aracaju, Sergipe, Brazil; 9 DDS, PhD, Professor. Department of Oral Surgery and Pathology, School of Dentistry, Universidade Federal de Minas Gerais (UFMG), Belo Horizonte, MG, Brazil; 10 DDS, PhD, Professor. Oral Pathology Section, Department of Oral Diagnosis, Piracicaba Dental School, University of Campinas (UNICAMP), SP, Brazil; 11 DDS, PhD, Professor. School of Dentistry, Rio de Janeiro State University (UERJ), RJ, Brazil

## Abstract

**Background:**

Melanoma is an aggressive malignant tumor, rarely observed in the oral cavity. The aim of this study was to describe the clinicopathologic features of a series of oral melanomas.

**Material and Methods:**

A retrospective descriptive study was performed. A total of 15,482 biopsy records from two oral and maxillofacial pathology services in Brazil were analyzed. All cases of oral melanomas were reviewed, and clinical, demographic, histopathological data, treatment, and follow-up status were collected. In addition, immunohistochemistry stains (pan-cytokeratin AE1/AE3, vimentin, α-SMA, CD45, S-100 protein, HMB-45, Melan A, and Ki-67) were performed.

**Results:**

The series comprised of 5 males (71.4%) and 2 females (28.6%), with a mean age of 58.0 ± 9.2 years (range: 45-69 years) and a 2.5:1 male-to-female ratio. The gingiva (n = 3, 42.8%) and hard palate (n = 2, 28.6%) were the most common affected sites, presenting clinically as ulcerated swellings with a brown to black color. Cervical lymph node metastasis was detected in three patients during the first examination. Microscopically, 6 cases (85.7%) were melanotic, and one (14.3%) was amelanotic. Most cases (n = 4, 57.1%) presented a predominance of epithelioid cells. S-100 and HMB-45 were positive in all cases (n = 7, 100.0%). In contrast, only 4 cases (57.1%) were positive for Melan-A. The proliferative index with Ki-67 was high, with labeling index ranging from 70.0% to more than 90% of positive cells. Five patients died from complications of the tumors after a mean follow-up period of 7.8 months.

**Conclusions:**

Melanoma is an aggressive malignant tumor that rarely occurs in the oral cavity. It occurs mainly in adult and elderly patients and often is diagnosed in advanced stages. The current findings were similar to previous studies and reflected the characteristics of the services from where lesions were retrieved.

** Key words:**Head and neck cancer, melanoma, oral melanoma, oral mucosa.

## Introduction

Primary melanoma of the oral cavity is rare and corresponds to 2 to 8% of all melanomas (1) and about 0.5% of all malignancies of the oral cavity, with an incidence of 1.2 cases per 10 million individuals per year (2). Oral melanoma (OM) tends to occur in adults between the fifth and sixth decades of life. The upper alveolar ridge and hard palate are the most common intraoral site of occurrence (3).

In recent years, there has been a significant improvement in the survival of patients with melanoma, mainly due to the early detection of the tumor and new immunotherapeutic drugs (4). However, when melanoma occurs in the oral cavity, lesions in the early stages can mimic the clinical appearance of several other conditions making clinical diagnosis difficult (5), what contributes to that OMs are usually diagnosed in advanced stages, worsening the prognosis and contributing to the high mortality rates of this cancer (1,5).

In contrast to cutaneous melanoma (CM), the pathogenesis of OM is poorly understood. It is believed that OM originates from the malignant transformation of melanocytic cells present in the oral mucosa epithelium (5,6). There are no etiological factors associated with the development of OM; however, an association with BRAF, RAS, KIT, and BAP1 mutations have been described (7,8).

There are few Brazilian studies published in the English-language literature on OM to date (1,4,9-11), especially multi-institutional studies conducted in oral pathology services. Thus, this study aims to describe the clinicopathologic, and immunohistochemical features of 7 new cases of OM retrieved from two centers of oral pathology and medicine located in Brazil and to compare the findings with epidemiological data from different geographic locations.

## Material and Methods

- Patient samples and data collection

Cases diagnosed as oral melanomas were retrieved from the files of 2 Brazilian Oral Pathology Services: Department of Oral Diagnosis and Pathology, School of Dentistry, Federal University of Rio de Janeiro (UFRJ), Rio de Janeiro, Brazil; and School of Dentistry, Tiradentes University (UNIT), Aracaju, Sergipe, Brazil. Clinical data were retrieved from the pathology reports, and follow-up information (when available) was obtained from the referring physicians. Five-micrometer hematoxylin and eosin-stained sections were obtained from each case. Two oral pathologists (B.A.B.A and J.L.S.C) re-evaluated the histological features of the lesions.

- Immunohistochemistry (IHC)

Immunohistochemical analysis was performed in 3-μm-thick sections mounted on silane-coated glass slides and later were deparaffinized and rehydrated in graded ethanol solutions. After antigen retrieval in a pressure cooker with citrate buffer (pH 6.0), endogenous peroxidase activity was blocked with 20% hydrogen peroxide (H2O2), using five cycles of 5 minutes each. The primary antibodies used in the lesions included S-100 protein (polyclonal, dilution 1:10.000), HMB-45 (clone HMB-45, dilution 1:200), Melan A (clone A103, dilution 1:800), pan-cytokeratin (clone AE1/AE3, dilution 1:400), vimentin (clone Vim 3B4, dilution 1:400), α-SMA (clone 1A4, dilution 1:400), CD45 LCA (clone 2B11+PD7/26, dilution 1:200), and Ki-67 (clone MIB-1, dilution 1:100). Overnight incubation with the primary antibodies diluted in bovine serum albumin was followed by incubation with the secondary antibody conjugated with polymer dextran marked with peroxidase (Dako EnVision Labeled Polymer; Dako). Reactions were developed with a solution containing 0.6 mg/mL 3,3′-diaminobenzidine tetrahydrochloride (Sigma, St. Louis, MO) and 0.01% H2O2, and counterstained with Carazzi's hematoxylin. Positive control sections were used for each antibody, and negative control was obtained by omitting the primary specific antibody. In some cases, due to the prominent presence of melanin, prior to the immunohistochemical reactions, bleaching of melanin with hydrogen peroxide was performed to use DAB as a chromogen (12).

All slides were scanned into high-resolution images with Aperio Scanscope CS Slide Scanner (Aperio Technologies Inc., Vista, California, USA). Nuclear Ki-67 expression was scored based on the percentage (%) of positive nuclei, assessed digitally with the Nuclear Algorithm (Aperio Technologies Inc.).

## Results

- Clinical features

In the present study, 7 cases of melanomas of the oral cavity were retrieved. Of which 6 (85.7%) were melanotic, and only one (14.3%) was amelanotic. The prevalence of melanoma was 0.05%, from a total of 15,482 diagnostics. Table 1 summarizes the clinical features of these 7 cases. The patients comprised five men (71.4%) and two women (28.6%), with a mean age of 58.0±9.2 years (range 45 to 69 years) and 2.5:1 male-to-female ratio. The gingiva was the most affected site (n = 3, 42.8%), followed by the hard palate (n = 2, 28.6%). Other locations included alveolar ridge (n = 1, 14.3%), and lower lip (n = 1, 14.3%).

**Figure 1:**
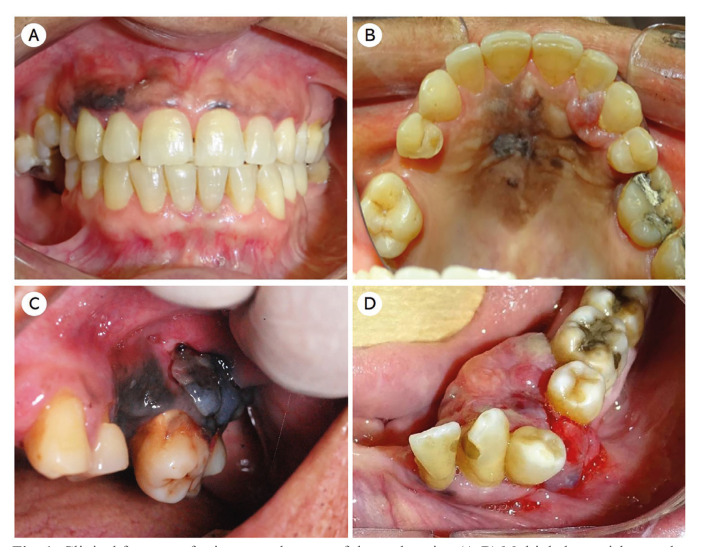
Clinical features of primary melanoma of the oral cavity. (A-B) Multiple brownish macules and dark-nodular lesions with an irregular shape, located on upper gingiva, between the right maxillary first premolar (#14) and the right canine tooth (#13) and in the hard palate (case 2). (C) Intraoral examination showing an ulcerated, blue-black, elevated multinodular lesion in the left posterior gingiva. The lesion extends across the hard palate and onto the attached vestibular gingiva of teeth 27 and 28 and vestibular sulcus (case 6). (D) A case of amelanotic melanoma presenting as a reddish-colored gingival growth located in the region between the lower left canine (#33) and the lower left second premolar (#35) (case 5).

Clinically, six cases (85.7%) presented as a nodular dark-colored swelling, firm to rubbery in consistency. One case (14.3%) showed reddish color, while in two cases, ulceration at the time of the first consultation was found (Fig. 1).

The size of the tumors ranged from 2 to 3 cm (mean 2.9±0.9). Most were asymptomatic (n = 5, 71.4%), although pain had been mentioned in some cases (n = 2, 28.6%). One patient complained of bleeding from the lesion. Three patients (42.9%) had lymph node involvement (regional metastasis) at the first examination. Of these, one patient also had distant metastases to the lungs and liver. Regarding the harmful habits of the patients, one (14.3%) reported being only a smoker, one (14.3%) referred to only alcohol use, and four (57.1%) informed both habits. In two cases (28.6%), information about smoking and drinking alcohol was not available.

Regarding clinical diagnosis, most cases had been diagnosed as melanoacanthoma and malignant neoplasms such as melanoma and Kaposi sarcoma. For the amelanotic lesion, the presumptive diagnoses included mainly benign proliferative processes such as pyogenic granuloma, peripheral giant cell lesion, and peripheral ossifying fibroma. All cases were submitted to the incisional biopsy.

The patients were treated with surgery, chemotherapy (paclitaxel and cisplatin) and/or radiotherapy. Outcome information was available from 5 patients (71.4%), with clinical follow-up ranging from 3 to 13 months (mean 7.8 months). Unfortunately, all patients died, and two lost to follow-up (Table 1).

**Table 1:**
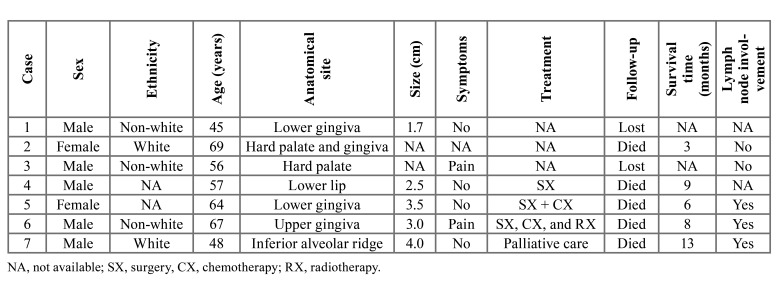
Summary of clinical features of 7 cases of oral melanomas.

In case 7, the patient was referred to an oncology service where imaging studies, such as chest radiograph and computed tomography, was performed and revealed hepatic and pulmonary metastases. TNM staging for the present case was stage IV (T4aN1bM1b) (13). Due to the advanced stage of the disease, the presence of multiple metastases, and the health state of the patient, no treatment was indicated, and he was subjected to palliative care.

- Pathologic and immunohistochemical features

**Figure 2:**
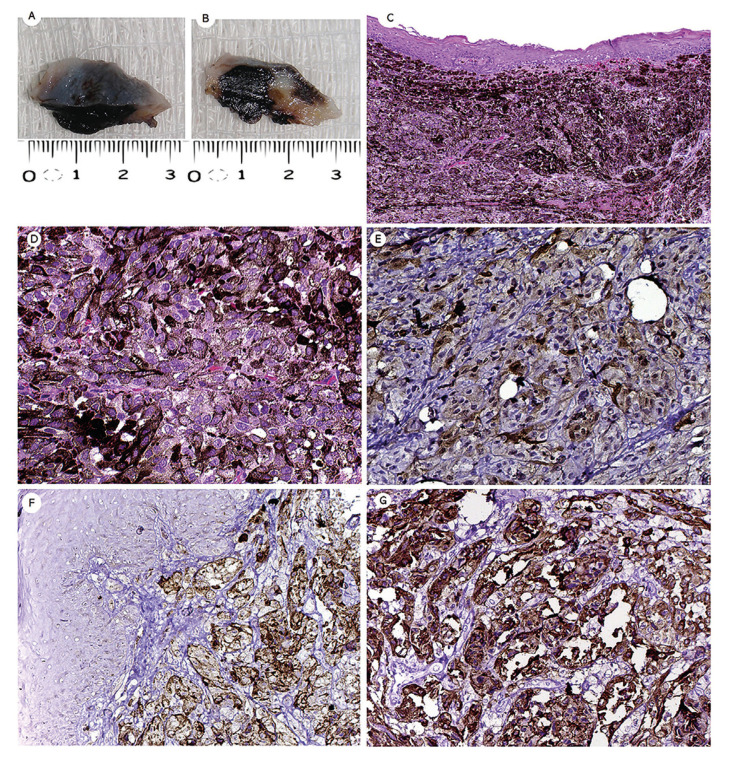
Gross appearance, microscopic, and immunohistochemical findings of oral melanoma (case 7). (A-B) Gross features of oral melanoma after an incisional biopsy. Detail of one ovoid soft tissue fragment was obtained, showing blue-black and brownish appearance. (C) The lesion was lined with parakeratinized stratified squamous epithelium. Note the evident junctional activity. (D) The cells and nuclei were markedly pleomorphic with amphophilic nucleoli and exhibited varying degrees of melanin pigments in the cytoplasm (hematoxylin and eosin stain, original magnification C ×100, D ×400). Tumor cells showed strong positivity for S-100 protein (E), HMB-45 (F), and Melan A (G) (IHQ, original magnification E, F, and G × 200).

On gross examination, the lesions were described as poor-circumscribed with smooth or irregular surface, soft to firm in consistency, and dark or brownish color and greasy cut surface (Fig. 2).

The amelanotic case (case 5) showed uniform yellowish color with a few dark or brownish areas and a greasy cut surface.

**Figure 3:**
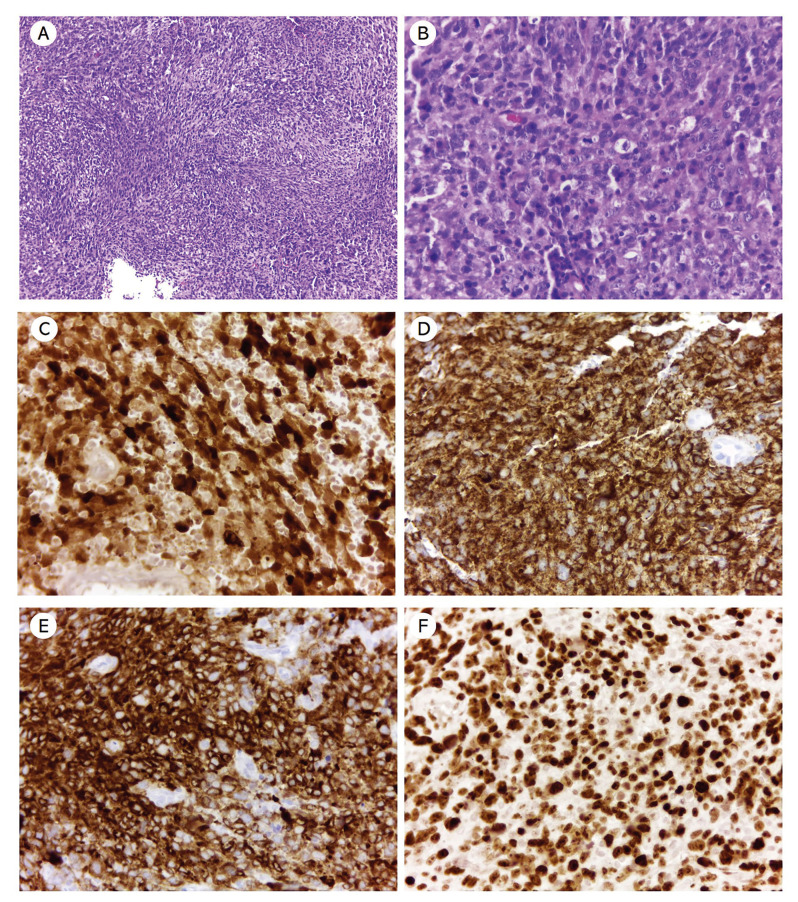
Microscopic and immunohistochemical features of oral amelanotic melanoma (case 5). (A-B) Oral melanoma showing mainly of amelanotic spindle and epithelioid melanocytes cells with a variable number of mitosis and absence of melanin deposition (hematoxylin and eosin stain, original magnification A ×100, B ×400). The immunohistochemistry results showed intense immunoreactivity for S-100 protein (C), Melan A (D), HMB-45 (E). Ki-67 (MIB-1) labeling index is approximately 90% (F) (IHQ, original magnification E, F, and G × 400).

**Figure 4:**
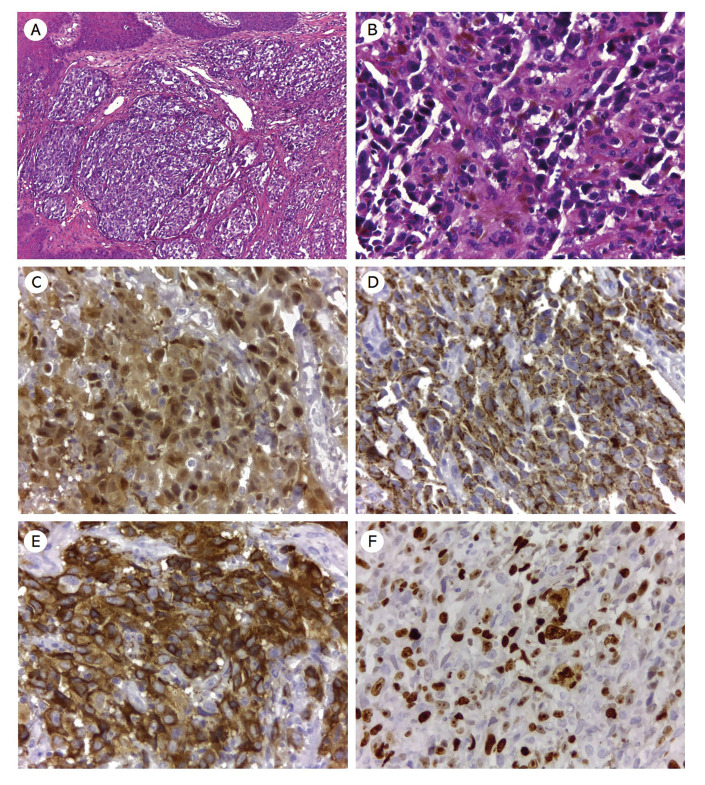
Microscopic and immunohistochemical features of oral amelanotic melanoma (case 3). Oral melanoma exhibiting a proliferation of spindle and epithelioid melanocytes organized in nests with a variable number of mitosis and (B) presence of focal melanin deposition (hematoxylin and eosin stain, original magnification A ×40, B ×400). The immunohistochemistry results showed strong nuclear and cytoplasmic positivity for S-100 protein (C), and intense immunoreactivity for Melan A (D) and HMB-45 (E). Ki-67 (MIB-1) labeling index is approximately 70% (F) (IHQ, original magnification E, F, and G × 400).

Histologically, all cases showed a diffuse proliferation of sheets and nests of pleomorphic melanocytic cells with epithelioid, rhabdoid, or spindle morphology arranged in an organoid pattern, with abundant melanin production. The tumor cells presented ovoid nuclei, pale cytoplasm containing brown granules compatible with melanin. Variable degrees of nuclear hyperchromatism and cellular pleomorphism were observed. The lesions were lined with parakeratinized stratified squamous epithelium exhibiting atypical and pleomorphic melanocytes in the basal and suprabasal layers (junctional activity). Atypical mitotic Figures were often seen. In addition, a scarce lymphocytic inflammatory response was present surrounding tumor cells (Fig. 2, Fig. 3, Fig. 4). Regarding cell morphology, 4 (57.1%) were predominantly epithelioid, 2 (28.6%) presented a predominance of undifferentiated cells, and 1 (14.3%) with predominant spindle cell morphology. Necrosis, perivascular, and perineural invasion was found in cases 3, 4, and 7, respectively. Morphological features of cases are illustrated in Fig. 2, Fig. 3, and Fig. 4. Carcinomas, sarcomas, and lymphomas were considered as the morphological differential diagnoses.

Immunohistochemical analysis was subsequently performed for confirmation of the phenotype of tumor cells. The tumor cells were strongly and diffusely positive in all cases for vimentin, S-100 protein, and HMB-45. Melan A was considered as focal/weak positive in 3 cases. In addition, all the cases were negative for α-SMA, pan-cytokeratin AE1/AE3, and anti-leukocyte common antigen (CD45). The Ki-67-positivity index was >70% in five cases, and two cases achieved more than 90%.

## Discussion

In the present study, the sample represented about 0.05% of the total lesions diagnosed in the referred services. Studies conducted in other pathology services reveal that OM also accounts for about 0.05% of all diagnosed lesions (6,9), which are data similar to our results.

The etiology of OMs remain unknown and seems to be different from CMs due to the lack of relation with risk factors, such as family history, association with atypical nevi, and exposure to ultraviolet radiation, conditions favoring the onset of melanoma of skin (3,8,9). Additionally, smoking has been suggested as a risk factor for OM because it is known that pigmented oral lesions are more prevalent among smokers. The present study demonstrated an association with tobacco use in 71.4% of cases. These facts would explain the occurrence of OMs in some cases reported herein since the patients had been exposed to the deleterious effects of smoking for many years. However, it is difficult to interpret the influence of the harmful habits on the development of OM due to the lack of data and/or absence of evaluation criteria in the literature.

OM has an average age at diagnosis of approximately 60 years and usually affects patients between the fifth and seventh decades of life, but a wide age range has been reported (14). Both females and males can be affected, although some studies report a female predominance (5,14). Others report slight male predominance (3,15-18), similar to this present series. However, in some studies, no predilection for sex is observed (19).

Clinically, individuals affected by OM most often exhibit an asymptomatic extensive irregular mucosal lesion that presents with nodular or macular areas, showing a blackish coloration resulting from the intense deposition of melanin pigments by neoplastic cells (20,21). Moreover, OM may present different color nuances in the same lesion, showing some areas with a darker coloration and others with pale, reddish, and/or brownish coloration (3,6). Other complaints such as ulceration, bleeding, pain, paresthesia, and tooth mobility may be observed. The hard palate and upper gingiva/alveolar ridge are the most affected sites by OM, similar to this present study; however, cases involving buccal mucosa, tongue, and floor of the mouth are reported (6,22). According to these data, in our study, in most patients, the clinical presentation was initially asymptomatic, and the patients sought a specialized service only after the significant growth of the lesion. Several cases published in the literature emphasize that due to the asymptomatic behavior of OMs in their initial phases, there is a frequent delay in the seek professional assistance, which contributes to the tumors to be often diagnosed in advanced stages, contributing to a poor prognosis (5,6).

Clinical differential diagnosis is extensive and includes a spectrum of reactive, benign, and malignant lesions with distinct biological behaviors (20,21). Additionally, foreign bodies, heavy metal intoxication, or drugs may also promote pigmented lesions in the oral cavity (3,20,21). These lesions are often detected by the patient or during a routine dental exam. The clinical history, symmetry, borders, and color uniformity are essential in determining the clinical differential diagnosis (3). However, amelanotic melanomas, such as case 5, present a challenge to clinical diagnosis. The absence of melanin pigmentation makes the clinical diagnosis of these tumors extremely challenging due to the clinical similarity with several other conditions that affect the oral cavity, including infectious diseases, non-neoplastic proliferative processes, reaction lesions, and other oral malignancies (1).

Histologically, OM is known for its distinct ability to mimic several neoplasms, because of its heterogeneous morphological features, besides the absence of melanin deposition seen in some cases (22,23). In this situation, the histopathologic differential diagnosis is wide. The morphological differential diagnosis includes spindle cell malignant tumors (such as leiomyosarcomas, malignant peripheral nerve sheath tumor, spindle cell carcinoma, undifferentiated pleomorphic sarcoma, angiosarcoma, synovial sarcoma), malignant epithelioid tumors (such as epithelioid sarcoma), round cell tumors (such as lymphomas, neuroendocrine tumors, rhabdomyosarcoma, plasmacytoma), clear cell malignant tumors (such as renal cell carcinoma, mucoepidermoid carcinoma), and metastatic lesions (3,5,6,23). Characteristically, OM cells exhibit prominent central, single, and amphophilic nucleoli. The neoplastic cells may be arranged in different architectural patterns, such as pagetoid, alveolar, organoid, fascicular, or storiform growths, or as a combination of these patterns. Extracellular and intracellular melanin can be present in varying quantities. Besides, perineural and perivascular invasions and necrosis may be seen (23). Most of these features were observed in the present series, supporting the diagnosis of melanoma. In our series, epithelioid morphology predominated in most cases. The type cell seems to have no impact on the prediction of tumor behavior (19,24); however, a recent study has indicated poor prognosis for tumors with epithelioid morphology (25). These factors may explain the aggressiveness of these tumors; however, other studies can confirm these early observations.

When melanin pigmentation is present, OM can usually be easily diagnosed by morphological analysis (6). However, when the lesion is amelanotic, such as in case 5, the immunohistochemistry analysis is essential to establish the correct diagnosis (1,5). In both situations, positive immunohistochemical reactions for S-100 protein, HMB-45, and Melan A confirm the diagnosis of OM (1,6,23), as occurred in our series.

OMs are aggressive neoplasms with difficult therapeutic management. The patients' clinical approach is usually determined by the presentation of the tumor, considering the surgical possibilities and the quality of life of the patients (26). OM treatment includes radical surgery, cryotherapy, chemotherapy, radiotherapy, or immunotherapy used individually or in combination (23,26). In general, the prognosis of patients with OM is very unfavorable, with survival rates lower than those described for patients with cutaneous melanoma (1,26). However, the causes for this difference in behavior between the two presentations remain unknown, and factors such as histopathological presentation, diagnostic delay, or anatomical complexity that prevent adequate surgical resection may be related.

Factors associated with a worse prognosis include tumor polymorphism, vascular invasion, advanced age, positive surgical margins, deep infiltration, and necrosis (6,8). Classical parameters to the prognosis of CM, such as Clark's level and Breslow's depth, have limited application to mucosal melanomas, maybe because most tumors are more profound than 4mm when diagnosed. Local recurrences are reported in more than 50% of cases, while 5-year survival for patients affected by OMs is as low as 15%, which makes early diagnosis essential (26).

Despite the classical clinical and morphological features in most cases of the present series, our patients were diagnosed with advanced disease. This is mainly attributed to late diagnosis (13). Thus, it is essential to perform a histopathological evaluation of any suspicious pigmented lesion since early diagnosis, and surgical removal are essential for a favorable prognosis contributing thus to the effective treatment of these lesions and cancer prevention (27).

In summary, the epidemiologic profile and clinical characteristics of OM were similar to those described in Brazil and other countries. Despite its rarity, OM should be considered in the differential diagnosis of pigmentated soft tissue lesions located in the oral cavity, principally in the gingiva, alveolar ridge, and palate of adult patients.
